# Active machine learning for transmembrane helix prediction

**DOI:** 10.1186/1471-2105-11-S1-S58

**Published:** 2010-01-18

**Authors:** Hatice U Osmanbeyoglu, Jessica A Wehner, Jaime G Carbonell, Madhavi K Ganapathiraju

**Affiliations:** 1 Department of Biomedical Informatics, University of Pittsburgh School of Medicine, Pittsburgh, PA, USA; 2 Department of Mathematics, University of North Carolina, Chapel Hill, NC, USA; 3 Language Technologies Institute, Carnegie Mellon University, Pittsburgh, PA, USA; 4 Intelligent Systems Program, University of Pittsburgh School of Art and Sciences, Pittsburgh, PA, USA

## Abstract

**Background:**

About 30% of genes code for membrane proteins, which are involved in a wide variety of crucial biological functions. Despite their importance, experimentally determined structures correspond to only about 1.7% of protein structures deposited in the Protein Data Bank due to the difficulty in crystallizing membrane proteins. Algorithms that can identify proteins whose high-resolution structure can aid in *predicting *the structure of many previously unresolved proteins are therefore of potentially high value. Active machine learning is a supervised machine learning approach which is suitable for this domain where there are a large number of sequences but only very few have known corresponding structures. In essence, active learning seeks to identify proteins whose structure, if revealed experimentally, is maximally predictive of others.

**Results:**

An active learning approach is presented for selection of a minimal set of proteins whose structures can aid in the determination of transmembrane helices for the remaining proteins. TMpro, an algorithm for high accuracy TM helix prediction we previously developed, is coupled with active learning. We show that with a well-designed selection procedure, high accuracy can be achieved with only few proteins. TMpro, trained with a single protein achieved an F-score of 94% on benchmark evaluation and 91% on *MPtopo *dataset, which correspond to the state-of-the-art accuracies on TM helix prediction that are achieved usually by training with over 100 training proteins.

**Conclusion:**

Active learning is suitable for bioinformatics applications, where manually characterized data are not a comprehensive representation of all possible data, and in fact can be a very sparse subset thereof. It aids in selection of data instances which when characterized experimentally can improve the accuracy of computational characterization of remaining raw data. The results presented here also demonstrate that the feature extraction method of TMpro is well designed, achieving a very good separation between TM and non TM segments.

## Background

Membrane proteins mediate a broad range of fundamental biological processes such as cell signaling, transport of molecules that cannot otherwise cross impermeable cell membranes, cell-cell communication, cell recognition and cell adhesion [1]. About 8000 (or 30%) of human genes encode for membrane proteins, the sequences of which are relatively easy to obtain. In contrast, of the 60,369 protein structures deposited to-date in the Protein Data Bank (PDB), only 1062 or 1.7% correspond to membrane proteins [2]. Production of membrane protein crystals which is required for determining highly accurate detail of the 3D structure is very difficult or sometimes impossible due to the inherently hydrophobic nature of membrane proteins [3,4]. NMR methods do not apply readily to very large molecules such as transmembrane proteins. Knowledge of the transmembrane (TM) segment locations in a membrane protein can narrow down possible tertiary structure conformations for the given protein [5-8] and aid in prediction of its function. Therefore prediction of the structure by computational methods is useful.

Labeling data (or characterizing) by experimental or other manual methods is often time consuming and expensive. To characterize data by computational methods, supervised machine learning approaches require a training set which is a representative sample of all the unlabeled data in order to achieve comparable performance on the latter. In practice, data selection for experimental or manual characterization is rarely ever carried out by taking into account the complete space spanned by the unlabeled data. It is useful and efficient to design algorithms that can not only learn from existing training data but can also direct the optimal selection of new data instances for manual labeling. Active learning, a type of supervised learning, samples the unlabeled pool of data and selects instances whose labels would prove to be most informative additions to the training set. Each time new labelled instances are added to the training set, the classification function is updated. As a consequence of this, the information valuable to the learning function is maximized.

Common strategies employed for data selection in active learning [9] are *density based*, where a set of data points from dense regions are selected for labelling [10,11]; or *uncertainty based*, where data points with maximum confusion or uncertainty with current classifier are selected [12,13]; or *representative based*, in which data points most representative of the data set are selected [14]; or *ensemble based *in which multiple criteria are employed [15-17]. Many of the active learning approaches combine density-based and uncertainty based strategies to achieve better performance. Clustering is commonly applied to select the representative data points [18-22].

The scarcity of labelled data for TM helix prediction makes it an excellent candidate for active learning. In the case of transmembrane helix prediction, unlabeled data refers to sequences of all the membrane proteins and labeling refers to determination of structural annotations by experimental means. Technological improvements over the last decade lead to a rapid increase in biological data including gene sequences from several organisms. One of the major challenges of bioinformatics relates to this flood of raw data. Moreover, in some cases such as transmembrane (TM) helix prediction, manual annotation is very difficult or sometimes impossible [4].

Early TM helix prediction methods use two fundamental characteristics: (i) the length of the TM helix being at least 20 residues so that it is long enough to cross the 30 Å thick lipid bilayer [23], and (ii) the TM residues being hydrophobic for reasons of thermodynamic stability in the hydrophobic membrane environment [7]. Explicit methods employ a numerical scale to code for the hydrophobic property of amino acids followed by computing average-hydrophobicity in moving windows, to locate long hydrophobic segments [24,25]. Other methods treat the 20 amino acids as distinct entities, without explicit representation of their similarities, and statistically model their distribution in different topological locations of TM proteins [26,27]. These methods generally use statistical modelling and assume that membrane proteins conform to the commonly observed topology of cytoplasmic-TM-extracellular. Drawbacks with these methods have been low accuracy with the windowing methods and over-fitting by statistical methods due to the lack of sufficient training data. Recently, Ganapathiraju et. al. developed TMpro, a computational method with a radically different method for feature computation that attains a good separation of the TM versus non-TM "windows" and thereby, a high accuracy in TM helix prediction [28].

Here, TMpro is employed for transmembrane helix prediction, in conjunction with active learning algorithms to select a minimal set of proteins to train the TMpro statistical models (henceforth called TMpro-active).

## Methods

### Datasets

Prior to TMpro, TMHMM has been the most widely-used transmembrane helix prediction algorithm. A set of 160 proteins was used to train TMHMM [29]. In order to compare the performance of TMpro with TMHMM, in [28] the same training dataset of 160 proteins has been used to train TMpro. Keeping the training dataset the same, evaluations were presented on three datasets: benchmark data, PDB_TM and MPtopo. To demonstrate the merits of active learning in comparison to non-active learning based methods, evaluations of TMpro-active are presented in comparison to TMpro without active learning. Note that, when all the data in training, namely all of the 160 proteins are selected, TMpro-active is the same as TMpro.

The following three datasets of membrane proteins with high resolution structural annotations are used for evaluation: (i) high resolution set from results reported by the benchmark evaluation by Chen et. al [30], (ii) membrane proteins with high resolution information from the MPtopo dataset consisting of 101 proteins and 443 TM segments in [31], (iii) PDBTM dataset downloaded in April 2006 (in order to compare the results with what have been published before [28], an older dataset is used); it contains all transmembrane proteins with 3D structures from the PDB determined to that date [2]. PDBTM provides a non-redundant subset of alpha-helical TM proteins having sequence identity less than 40%. Chains corresponding to this non-redundant list were extracted from the complete set, resulting in 191 proteins consisting of 789 TM segments [2]. 12 out of 191 proteins of PDBTM and 16 out of 101 proteins of MPtopo are redundant with the training set. A 2-class labeling scheme has been employed for all three datasets where each residue is marked as "non-TM" (inside and outside regions of the membrane) or "TM".

### Approach

The proposed approach, TMpro-active, explores the feature space to identify the data points, and thereby the proteins, which are most-representative of the feature space. Proteins thus selected are used to train the neural network of the original TMpro algorithm. The steps involved in TMpro-active are as follows (steps in italics are new compared to TMpro):

(1) To begin with, all proteins are considered to be unlabeled proteins.

(2) Primary sequence of each protein is expanded into five different primary sequences, each coding for one of polarity, charge, aromaticity, size and electronic property.

(3) Features are computed over moving windows of length 16 as done for TMpro.

(4) A Self-Organizing-Map is constructed over the feature space spanned by the proteins.

(5) Active learning algorithm is applied to determine the training set iteratively.

(6) A neural network (NN) is trained with the selected training set, and the output of the neural network is interpreted as done for TMpro.

### Feature computation

This process is same as carried for TMpro, but is presented here for completeness of information.

### Data preprocessing and vector space representation

The primary sequence of each protein is decomposed into five different sequences by replacing each amino acid with its property according to charge, polarity, aromaticity, size and electronic property [28]. The protein sequence, of length L, is analyzed with a moving window of length *l*; the window is moved along the sequence one residue at a time, each position of the window yielding a feature vector. The feature vector at position *i*, represented by *R*_*i*_, is derived from the window beginning at the *i*^th ^residue and extending *l *residues to its right. It is given as(1)

where *C*_*ij *_is the count of property-value *j *in window *i*. The specific property-values counted by the *C*_*ij *_are as follows:

C_i1_: count of "charge-positive"

C_i2_: count of "charge-negative"

C_i3_: count of "charge-neutral"

C_i4_: count of "polarity-polar"

C_i5_: count of "polarity-nonpolar"

C_i6_: count of "aromaticity-aromatic"

C_i7_: count of "aromaticity-aliphatic"

C_i8_: count of "aromaticity-neither"

C_i9_: count of "electronic property-strong acceptor"

C_i10_: count of "electronic property-strong donor"

C_i11_: count of "electronic property-acceptor"

C_i12_: count of "electronic property-donor"

C_i13_: count of "electronic property-neutral"

C_i14_: count of "size-medium"

C_i15_: count of "size-small"

C_i16_: count of "size-big"

When feature vectors *R*_*i *_are computed for every position of the window, moving to the right one residue at a time, the entire protein will have a matrix representation *p *(Equation 2),(2)

whose columns are the transpose of feature vectors *R*_*i *_(Equation 1).

### Singular value decomposition

Amino acid properties for feature representation (C_i,1 _to C_i,16_) are mutually dependent. It is therefore desirable to transform these feature vectors into an orthogonal space prior to the use of this data for clustering and features for prediction. To achieve this, protein feature matrices of all the proteins are concatenated to form a large matrix *A*, and subjected to singular value decomposition (SVD)(3)

where *U *and *V *are the right and left singular matrices and S is a diagonal matrix whose elements are the square roots of the eigen values of the matrix *AA*^*T*^. Only the top 4 dimensions have been used for feature presentation, since the top 4 dimensions of S of training data have been found to carry 85% of the energy (variance) [28]. The matrices *U*, *S *and *V *are dependent on the matrix *A *from which they are computed.

Therefore, for each new protein, singular value decomposition should ideally be recomputed. However, this would also involve recomputation of all the statistical models built on the features derived through singular value decomposition. To avoid this, the feature vectors along the same principal components can be approximated by multiplication *R*_*i *_with *U*^*T *^similarly as given in Equation 3 [28].

### Data clustering

A Self-Organizing Map (SOM) is computed as a way of clustering the unlabeled data. It arranges a grid of nodes topologically based on the values of the features of the training data, such that adjacent nodes are more similar to each other. The SOM grid can efficiently be used to visualize and investigate properties of the data [32]. In basic SOM, the neurons (nodes) are located on a low dimensional grid, usually one- or two-dimensional. The basic SOM construction follows an iterative procedure. Each neuron receives the input signal vectors weighted by a vector indicating the degree of closeness of peripheral neurons. For each input vector, the node closest to it is determined via similarity between the input vector and the weights of each node. In the training process, the weights of the winning neuron and its adjacent neurons are updated in terms of distance of each of these neurons from the input vector.

**Figure 1:**
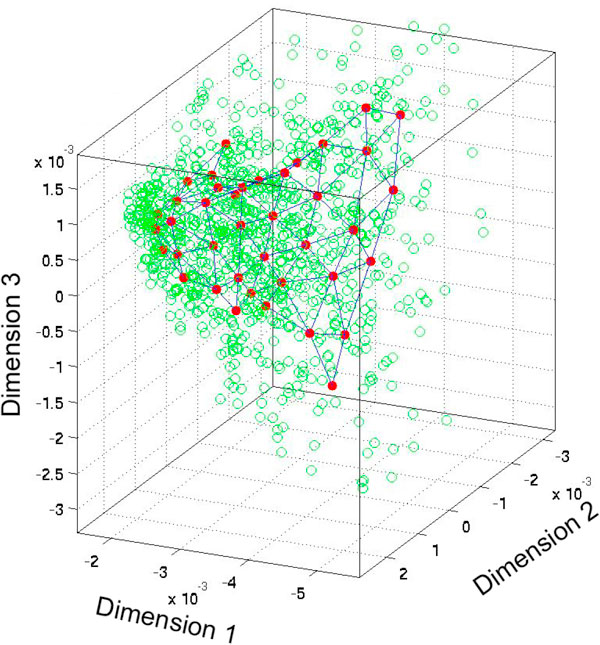
**The coverage of SOM network over the data**. Figure represents the coverage of the SOM network. 1000 data points are just shown for more clear representation.

An SOM has been created with 50 nodes arranged in 5 × 10 hexagonal lattice grid [33], and is trained on 1000 random data points (see Figure 1). The hexagonal lattice emphasizes the diagonal directions in addition to horizontal and vertical directions (see [32] for details). Euclidian distance is used to calculate distance between nodes to its neighbours. Weights of nodes are used as cluster centroids. The SOM is then *simulated *for the entire set features - which amounts to clustering of the data around these 50 nodes.

### Active learning

As discussed earlier, active learning is an approach to minimize the number of labelled proteins that form the training set. The algorithm can actively choose a minimal training set. To explore design choices that affect this desirable behaviour, four different experiments were designed for choosing the training set. We have a pool of 160 proteins that are considered to be unlabeled by the algorithm, from which it can select training proteins.

#### Random selection

This method is considered the baseline with which to compare active learning techniques. One protein is selected randomly per iteration and added to the training set.

#### Selection by node-coverage

Coverage of the feature space spanned by a protein is indicated by the nodes of the SOM to which the features are assigned. All proteins in the data set are ranked by the number of nodes they each cover; i.e., by the number of nodes into which their features fall. The protein that covers the largest number of the nodes of the SOM is selected and added to the training set.

#### Selection by maximal entropy (confusion-rated)

Initially, 1 protein is selected randomly and is added to the training set. Subsequent to that, in each iteration, proteins whose feature vectors fall in the nodes with maximum confusion between TM and non-TM labels, or rather nodes with higher confusion rate as determined by their entropy, are selected and added to the training set. The confusion (entropy) in labelling the data points can be measured as(4)

where p_0 _is the fraction of data points labelled TM and p_1 _is the fraction of data points labelled non-TM. By choosing proteins from highest confusion nodes, we assume that the most informative proteins are those that the classifier is most uncertain about.

#### Selection by both node-coverage and confusion-rate

Proteins whose labels are asked are selected according to node coverage and confusion rate in an alternating manner in each iteration.

### Neural networks for feature classification

In each iteration, the neural network classifier is retrained with the updated set of labelled proteins, and prediction performance on test data is evaluated. The neural network is modelled as described before [22]. During training, a class label is defined for each window based on the number of TM and non-TM residue labels in the window:

• Completely-membrane (Class label = 1): If all residues in the window are labeled TM

• Completely-nonmembrane (Class label = -1): If all residues in the window are labeled non-TM

• Mixed (Class label = 0): If some residues in the window are labeled TM and some non-TM

The number of input nodes of the NN is 4 and the number of output neurons is 1. One hidden layer of 4 nodes is placed in between input and output layers. The model is fully connected in the forward direction. Each of the hidden and output neurons is a tansig classifier [33]. Back-propagation procedure [33] is used to train the network by presenting it with feature vectors and their corresponding target output class labels. Mixed label feature vectors are not presented for training, since they arise from both TM and non-TM residues and hence are expected to lie in the "confusable" region in the features space. The output neuron learns to fire -1 for non-TM features and +1 for TM features. A threshold of 0.4 is chosen for automatic classification of the feature into its class. Each input feature vector causes the output neuron to fire an analog value ranging from -1 (non-TM class) to +1 (TM class). A threshold of 0.4 is used to label the residue at the first position in the window to be TM or non-TM. Since the feature is derived over a window of length 16, and threshold of 0.4 is "more confident" towards the TM label, the 8 residues starting from the first position of the window are all set to be of TM type (these numbers are heuristically chosen during cross validation). The process is repeated for the next feature vector, and so on, and a TM label is assigned to 8 residues at a time every time the output neuron fires a value greater than the threshold. Evaluation is carried out with the same metrics as designed by Chen et al in [30]. The same metrics were also used in comparing TMpro with TMHMM in [28].

### Implementation

Singular value decomposition of the protein feature matrix is computed using the SVDS tool in MATLAB^®^. The SOM Toolbox of MATLAB^® ^is used to create SOMs. Training and classification procedures for neural networks are implemented using the Neural Net toolbox of MATLAB^®^.

## Results and discussion

### Self organizing map

SOM is implemented to cluster the unlabeled data. Only 1000 random data points are used while training the SOM network. After 1000 random data points, increasing the amount of unlabelled data did not improve clustering efficiency. Moreover, by using a small amount of data points, SOM can be formed as quickly as possible. Figure 1 shows the resulting SOM among the spread of a random sample of unlabeled data. Not all unlabeled data is shown so as to keep the SOM nodes visible.

### Benchmark analysis of transmembrane segment prediction in membrane proteins

Predictions are uploaded to the transmembrane helix (TMH) benchmark evaluation server [27]. Transmembrane helix (TMH) benchmark server is an excellent resource to quantitatively compare new TM helix prediction methods with previous methods which include both simple hydrophobicity scale methods to more advanced algorithms that use hidden Markov models, neural nets, etc. The benchmark server uses the following metrics for evaluations [30]: Q_*ok *_is the percentage of proteins whose membrane segments are all predicted correctly. Segment-recall (called *Q*_*obs*_^*htm *^on benchmark server) is the percentage of experimentally determined (or 'observed') segments that are predicted correctly. Segment-precision (called Q_*pred*_^*htm *^on benchmark server) is the percentage of predicted segments that are correct. The residue accuracy Q_2 _refers to the percentage of residues that are predicted correctly. We also computed the F-score, which is the geometric mean of segment level recall and precision. Since recall and precision can each be increased arbitrarily at the expense of the other, the two metrics when viewed independently do not reflect the strength of the algorithm. Hence, the geometric mean of the two, (effectively the point where the two measures are expected to be equal) is used as the metric.

As described in Methods section, *Random *selects proteins to add to the training set based on random selection, Node-coverage selects proteins that cover the largest number of nodes, Confusion-rated selects proteins that cover nodes with maximum confusion and Node-coverage & Confusion-rated alternately selects proteins based on node coverage and confusion-rated. In each iteration, the neural network is reinitialized and then trained with the updated labelled (training) data. This process is performed independently for each method and their performance is evaluated on the test data. Neural network initialization, training and evaluation are carried out ten times, and average performance over ten experiments is reported here. Table 1 shows the average performance for each of the learning algorithms reported by the benchmark server (on dataset 1) after first, second, fifth and tenth round of training. Active learning methods (Node-coverage, Confusion-rated and Node-coverage & Confusion-rated) outperformed Random selection (a passive learning algorithm). The results for the active learning method that performed best, Node-coverage, reached 94% segment level F-score even for a single protein with balanced performance between segment-recall (97%) and segment-precision (92%). Node-coverage & Confusion-rated performed similarly to Node-coverage after one protein, likely because this alternating method used Node-coverage selection on the first round rather than Confusion-rated. Compare this to Confusion-rated alone, which reached 91% segment level F-score only after a second iteration, and Random which only achieved 63% F-score for comparable size of training set. Active learning significantly reduced the cost (number of proteins labeled out of 160 proteins), while reaching high prediction accuracy, from over 100 training proteins for other state of the art TM helix prediction algorithms to only one training protein. Moreover, by using the smallest number of examples, a classifier can be trained as quickly as possible.

**Table 1:** Comparison of TMpro NN: applying active vs. passive learning algorithms for updating training set from benchmark analysis.

	Methods	# of Proteins in Training-Set	Qok	Qhtm	Qhtm	Qhtm	Q2
				Fscore	%obs	%prd	
1	Random	1	14	27	29	25	55
		2	36	63	67	60	65
		5	51	82	84	80	70
		10	54	91	95	88	73
2	Node-Coverage	1	61	94	97	92	75
		2	61	94	97	91	75
		5	63	94	97	92	75
		10	61	94	97	92	75
3	Confusion-Rated	1	14	27	29	25	55
		2	52	91	95	87	73
		5	55	91	95	88	73
		10	59	93	96	89	74
4	Node-Coverage & Confusion-Rated	1	61	94	97	92	75
		2	59	92	96	88	73
		5	58	92	96	89	73
		10	61	94	96	91	74

It can be seen that TMpro achieves high segment accuracy (F-score) even if the classifier is trained with just one protein that is found by Active Learning algorithms. The columns from left to right show: method being evaluated; Number of proteins in training-set; Protein level accuracies: Qok, which is the percentage of proteins in which all experimentally determined segments are predicted correctly, and no extra segments are predicted; that is, there is a one to one match between predicted and experimentally determined segments; Segment F-score which is the geometric mean of Recall and Precision; Recall (Qhtm,%obs, percentage of experimentally determined segments that are predicted correctly); and Precision (Qhtm,%pred percentage of predicted segments that are correct). Q2 is the residue level accuracy when all residues in a protein are considered together, and the Q2 value for the entire set of proteins is the average of that of individual proteins. See [30]for further details on these metrics.

TMpro-active shows similar performance over TMpro. However, lower Q_ok _is observed, which is likely due the smaller number of training proteins. When the training data includes larger number of proteins, TMpro-active would be the same as TMpro in algorithm (see [28] for performance evaluation of TMpro by training with all of the 160 proteins).

### Performance on MPtopo and PDBTM data sets

**Figure 2:**
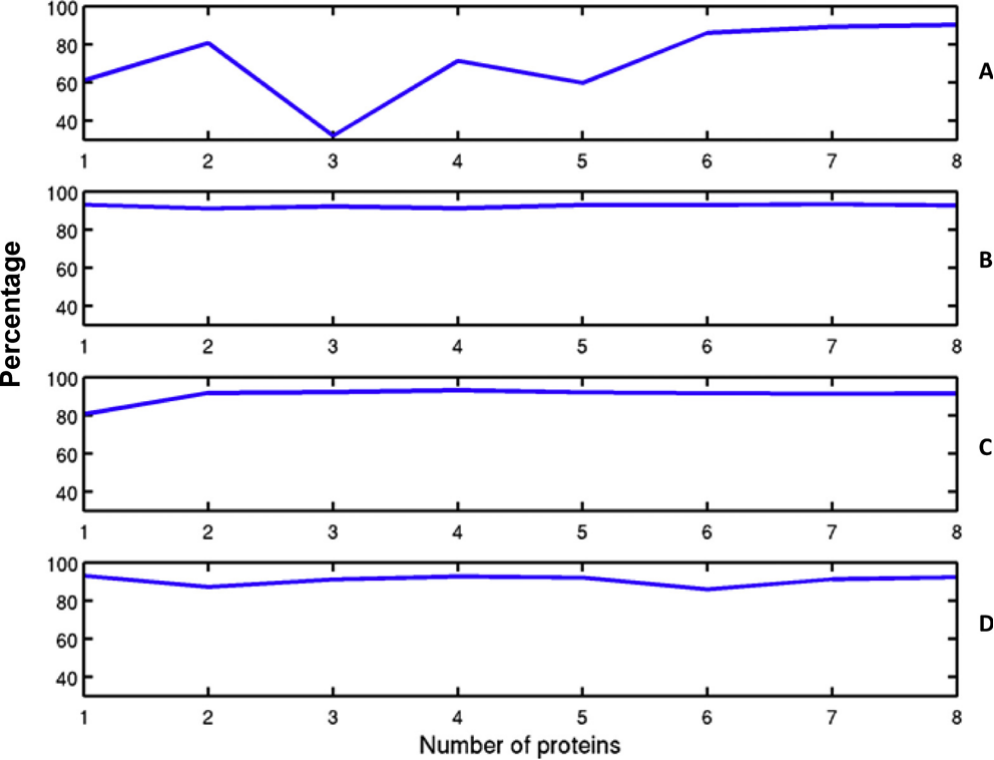
**Segment level TM prediction F-score results for MPtopo**. (A) Random, (B) Node-coverage, (C) Confusion-rated, (D) Node-coverage and confusion-rated. It can be seen that TMpro achieves high segment accuracy (F-score) even if the classifier is trained with just one protein that is found by active learning algorithms. Node-Coverage shows best performance.

We additionally tested prediction performance of our active learning algorithms on two larger data sets, MPtopo dataset of 101 proteins (see results in Table 2) and PDBTM dataset of 191 proteins (see Table 3). Figure 2 shows comparison of F-scores for Active Learning. For both data sets, Active learning methods outperformed random selection. The results for the active learning method that performed best for MPtopo and PDBTM is Node Coverage. This method reaches on average 91% segment level F-score even for single protein with balanced segment recall (92%) and precision (%90) on MPtopo data set. Moreover, when trained even with a single protein, it achieved an F-score of 91%, segment recall of 93% and segment precision of 90% on a PDBTM dataset. Although for second iteration the performance is reduced for both data sets, it recovered after tenth iteration. Confusion-rated and Node-coverage & Confusion-rated perform approximately the same after second iteration. Active learning significantly reduced the cost (number of proteins labeled out of 160 proteins) while reaching high prediction accuracy. As shown in Table 2 and Table 3, Node-coverage continued to outperform both Random and Confusion-rated, achieving about 91% segment F-score with only five training protein, as opposed to Random with 84% on MPtopo and 76% on PDBTM and Confusion-rated with 89% on MPtopo and 85% on PDBTM. Again, Node-coverage & Confusion-rated performed the same as just Node-coverage on the first round, but on subsequent rounds the F-score decreased slightly below just Node-coverage, perhaps showing that Confusion-rated is known to typically exceed performance compared to density-based selection after acquiring some amount of initial training [34]. In this case however, the feature creation has been very effective and the learning rate is significantly high even with a single protein, leaving no room for confusion rated selection to overtake density based selection.

**Table 2:** Comparison of TMpro NN: applying active vs. passive learning algorithms for updating training set from MPtopo (101 proteins, 443 TM segments).

	Methods	# of Proteins in Training-Set	Qok	Qhtm	Qhtm	Qhtm	Q2
				Fscore	%obs	%prd	
1	Random	1	14	40	41	41	63
		2	25	60	59	61	67
		5	36	84	88	81	74
		10	35	79	81	78	74
2	Node-Coverage	1	44	91	92	90	78
		2	44	91	91	90	79
		5	44	91	92	90	79
		10	46	91	92	90	79
3	Confusion-Rated	1	26	68	75	65	67
		2	34	85	90	81	75
		5	41	89	91	87	78
		10	45	91	91	90	79
4	Node-Coverage & Confusion-Rated	1	44	91	92	90	78
		2	44	90	91	89	79
		5	44	91	91	90	79
		10	45	90	91	90	78

For description of columns, see caption of Table 1. Q_htm_^%obs ^and Q_htm_^%pred ^have been computed per-protein and averaged over all the proteins.

**Table 3:** Comparison of TMpro NN: applying active vs. passive learning algorithms for updating training set from PDBTM (191 proteins, 789 TM segments).

	Methods	# of Proteins in Training-Set	Qok	Qhtm	Qhtm	Qhtm	Q2
				Fscore	%obs	%prd	
1	Random	1	20	51	54	49	20
		2	32	69	72	66	32
		5	35	76	78	73	35
		10	35	78	81	75	35
2	Node-Coverage	1	50	91	93	90	79
		2	47	90	91	88	79
		5	49	91	92	89	79
		10	50	91	93	90	79
3	Confusion-Rated	1	20	48	51	46	70
		2	36	81	84	78	75
		5	38	85	90	81	74
		10	46	90	92	87	78
4	Node-Coverage & Confusion-Rated	1	50	91	93	90	79
		2	49	90	92	88	78
		5	48	91	93	89	79
		10	51	91	93	90	79

For description of columns, see caption of Table 1. Qhtm,%obs and Qhtm,%pred have been computed per-protein and averaged over all the proteins.

## Conclusion

Active learning is a promising method for bioinformatics applications such as membrane structure prediction and protein-protein interaction prediction which are marked by availability of small amounts of fully-characterized data and unwieldy procedures for experimental characterization. In this paper, active learning has been employed to tag the proteins that prove to be most informative in training a transmembrane-helix prediction algorithm, TMpro. Results show that active learning can significantly reduce the labelling costs without degrading performance. It is seen that *latent semantic analysis *of protein sequences [28,35] is highly effective for prediction of TM segments.

## Competing interests

The authors declare that they have no competing interests.

## Authors' contributions

MKG designed the algorithms. JAW developed the framework for feature analysis and clustering and HUO developed the active learning components. Manuscript has been prepared by HUO and MKG. JGC provided advice on algorithm-design and reviewed the manuscript.
